# Salicylate- and Noise-induced Tinnitus. Different Mechanisms Producing the same Result? An Experimental Model

**DOI:** 10.1007/s12070-023-04049-w

**Published:** 2023-07-04

**Authors:** Pavlos Pavlidis, Kyriaki Papadopoulou, Vasilis Spyridon Tseriotis, Sophia Karachrysafi, Chrysanthi Sardeli, Haralampos Gouveris, Theodora Papamitsou, Antonia Sioga, Dimitrios Kouvelas

**Affiliations:** 1 Department of Otorhinolaryngology / Head & Neck Surgery, University Medical Center Mainz, Mainz, Germany; 2 Laboratory for Clinical Pharmacology, School of Medicine, Faculty of Health Sciences, Aristotle University Thessaloniki, Thessaloniki, Greece; 3 Laboratory of Histology-Embryology, School of Medicine, Faculty of Health Sciences, Aristotle University of Thessaloniki, Thessaloniki, Greece

**Keywords:** Salicylate, NMDA receptor, spiral ganglion neurons, tinnitus, noise

## Abstract

Purpose: Tinnitus, the generation of phantom sounds, can be the result of noise exposure, however, understanding of its underlying mechanisms is limited. Purpose of the study was is to determine whether different concentrations of salicylate can cause tinnitus of different intensity. Methods: For the purposes of this study 50 male Wistar rats were used. The animals were divided into 5 groups (10 rats in each group). The animals that did not receive any substance were allocated to the control group (Group A). The second group (Group B) of rats received salicylate (Sigma Aldrich) intraperitoneally for 7 days (300 mg/Kg/day). The 3rd group (Group C) received salicylate intraperitoneally for 7 days, but at twice the concentration of the animals in the second group (600 mg/kg/d). The 4th group (Group D) simultaneously received salicylate (300 mg/Kg/day) and pure Memantine (Sigma Aldrich, 10 mg/kg/d) intraperitoneally for 7 days. The 5th group (Group E) did not receive any substance but was exposed for 168 consecutive hours (7 days) to sound to induce tinnitus. Cochlear activity was evaluated with the use of Distortion Product Otoacoustic Emissions (DPOAEs). At the end of the experimental period, the animals were sacrificed, and the right cochlea was removed and prepared for further histological and immunohistochemical studies. Results: The DPOAEs of animals treated either with salicylate as monotherapy or salicylate combined with memantine were indistinguishable from the noise floor, did not differ significantly compared to the animals of the control group or those expose to constant noise. The cochlear structures of Group E remained anatomically and functionally unaffected from the exposure to constant noise. Memantine does not seem to offer substantial protection to the cochlear structures, according to histological examination and hearing tests, however, the rats receiving it exhibited better results in behavioral tests. Conclusions: The administration of memantine does not contribute significantly to the reduction of tinnitus.

## Introduction

More than a century ago, at an early stage of its clinical use, salicylate was found to cause hearing loss and tinnitus in humans [1, 2]. Later, large doses of salicylate have been shown to induce tinnitus in animals [3, 4]. Salicylate-induced tinnitus is characterized both in humans and animals by its replicability and reversibility. Due to the high reliability of salicylate-induced tinnitus, this medication has been considered a very powerful and useful tool to experimentally induce tinnitus in animals, thus providing a calibrated way to study some aspects of the pathophysiology of tinnitus [5–7]. Salicylate has thus been the preferred way to induce tinnitus in the context of biomedical research for several decades [8, 9]. While salicylate-induced hearing loss is due to the competition of this medication with the cytoplasmic chloride’s contribution to the nonlinear capacitance of outer hair cells [9–11], the details of the mechanisms of salicylate induced tinnitus have only recently been elucidated [12, 13].

The NMDA receptor is a type of ionotropic glutamate receptor and its subunits have been identified: NR1 subunit, a family of four NR2 subunits (NR2A, NR2B, NR2C, NR2D), and two NR3 subunits [14]. The functional NMDA receptor is formed as a heteromer between an NR1 subunit and the NR2A and/or NR2B subunits. It has been proven that the receptor channel pore, which is permeable with K^+^, Na^+^, and Ca^2+^, is created by NR1 and NR2 subunits [15]. Instead of forming a functional NMDA receptor alone, NR3 subunits can only co-assemble with NR1 and NR2 subunits into receptor complexes [16, 17]. It is considered that the presence of an NR1 subunit is obligatory for a functional NMDA receptor [18, 19].

It is known that the function of central auditory neurons is modulated by salicylate via different pathways. Salicylate has no effect on glutamate evoked currents in the hippocampus, but significantly reduces the GABAA receptor currents in a concentration-dependent manner [20].

All the above findings, obtained from experimental and clinical studies, were the incentive to conduct this study. The first question this study aimed to answer is to determine whether different concentrations of salicylate can cause tinnitus of different intensity. The second question was about whether the simultaneous administration of memantine can limit the occurrence of tinnitus, or not. The final question investigated dealt with whether tinnitus induced by high-intensity sound has similar audiological and histological findings to those of tinnitus induced after administration of medications.

## Materials and Methods

### Animals

In total, 50 male Wistar rats with documented dates of birth (8 weeks old) were obtained through the Laboratory Animals’ Breeding Facility of the Veterinary School of the Aristotle University of Medicine and included in the study. All rats were housed with free access to food and water. No outer or middle ear pathologies were observed at baseline. The animals were divided into 5 groups (10 rats in each group). Ten animals that did not receive any substance were allocated to the control group (Group A). The second group (Group B) of rats received salicylate intraperitoneally for 7 days (300 mg/Kg/day). The 3rd group (Group C) received salicylate intraperitoneally for 7 days, but at twice the concentration of the animals in the second group (600 mg/kg/d). The 4th group (Group D) simultaneously received salicylate (300 mg/Kg/day) and pure Memantine (Sigma Aldrich, 10 mg/kg/d) intraperitoneally for 7 days. The 5th group (Group E) did not receive any substance but was exposed for 168 consecutive hours (7 days) to sound to induce tinnitus (16 kHz from a sound generator/Sine Random Generator Type 1027; Brüel & Kjær, Copenhagen, Denmark, amplifier R300 Plus Amplifier; Inter-M, Seoul, Korea) at 112 dB SPL. All behavioral tests were conducted during the activity period of animals (dark phase) at approximately the same time each day.

### Behavioral Tests

All animals were trained to perform an active avoidance task. Tests were performed in a conditioning box containing an electrical floor and a climbing pole. The conditioning paradigm consisted of dispatched sessions of 10 trials per session.

During conditioning, the sessions lasted 15–20 min. The conditioning stimulus was a 50 dB sound pressure level (SPL) pure tone with a frequency of 10 kHz of 3-sec duration. The unconditioned stimulus was a 3.7 mA electrical foot shock presented for 30 s at most. The time between the conditioned stimulus and the unconditioned stimulus was 1 s. Electrical shocks were stopped by the researcher conducting the tests when the animal correctly climbed. Intertrial intervals lasted at least 1 min. The score depicted the level of performance assessed by the number of times the rat correctly climbed in response to sound. Animals were deemed conditioned when the level of performance reached at least 80% in three consecutive sessions.

When conditioned (i.e., when animals scored at least 80% in three consecutive sessions), experimental animals were included in the experiments. The behavioral testing protocol consisted of daily measurement of true positive (correct) responses to sound and false positive responses. False positive responses represented the number of climbings during intertrial periods (i.e., responses during silent periods). If animals stayed on the pole for 10 s, they were removed and placed on the floor. Trials were randomized, and electrical foot shocks were only administered if the animals did not climb in response to sound. Whatever the true positive and false positive responses, each session included 10 trials and lasted 10 min. Both true and false positive responses were measured in the same session. In contrast to other protocols in which animals are deprived of food or water — which in some cases leading to major loss of body weight - this paradigm did not involve changes in the animals’ physiological states [21, 22].

### Distortion-Product Otoacoustic Emissions (DPOAEs)

The cochlear activity of the right ear of all rats was examined thrice during the experiment using DPOAEs (on day 0 = day before the experiment, day 7 and day 14, using the Vanderbilt protocol for DPOAE measurements. Two additional DPOAE-measurements were also obtained on days 7 and 14 after the discontinuation of the medication, to detect any possible delayed deterioration or improvement of the cochlear activity.

The DPOAEs at 2f1/f2 were drawn utilizing an ILO-96 cochlear emission analyzer (Otodynamics, London, UK). For DP-grams, the intensities of primary stimuli were set as equilevel (L1 = L2) at 65 dB. The frequencies (f1 and f2) were adjusted as f2/f1 = 1.21. The f2 frequencies examined for DP-grams ranged from 1 to 6.3 kHz (1001, 1184, 1416, 1685, 2002, 2380, 2832, 3369, 4004, 4761, 5652, 6299 Hz). The primary tones produced by two speakers were introduced into the animal’s outer ear canal through an inserted earphone probe. Detection threshold and suprathreshold measures in the form of input/output (I/O) functions were obtained by decreasing the primary tones from 75 to 36 dB SPL, in 3-dB steps. The DPOAEs were measured and recorded as an average of four separate spectral averages of each stimulus condition. The level of the noise floor was measured at the frequency that was 50 Hz above the DPOAE frequency, using similar averaging techniques [23, 24]. We have also measured the DPOAEs Input/output function (DP-I/O) was also measured in all rats. This function is recorded by measuring the DPOAE amplitude as a function of a change of stimulus level at a particular f2 frequency of 1000, 2000, 4000 and 6000 Hz. At each frequency level, the stimulus levels started at 75 dB for L1 and 65 dB for L2 then decreased in 5 dB until reaching for L1 and 40 Db FOR L2, as it has been described in previous studies [25].

### Preparation for Light Microscopy and Immunohistochemistry

Upon completion of the experiment, animals were sacrificed, and their cochleae were removed. and dissected into tissue blocks of 0.5 to 4.0 cm thickness and fixed by immersion into a 10% formalin solution (out of 35% formaldehyde stock solution). The specimens were then decalcified and dehydrated through an ascending series of alcohol solutions (76%, 96%, 100%, 100%). Subsequently, tissues were cleared into xylene for four hours. They were then dipped into liquid paraffin for an additional four hours so that embedding could follow. This was performed by placing the specimens into metallic molds, soaking them in liquid paraffin, and allowing them to cool at 4 ^0^ C for twenty minutes [26].

Afterward, sectioning of the paraffin blocks was performed using a semi-automated microtome at a thickness of 3 μm. Ten sagittal sections were collected by systematic random sampling. Three of these sections were placed on standard microscope slides, whilst the other seven were placed on seven positively charged slides. They were all allowed to dry at room temperature for one hour. The first two sections, destined for morphological analysis, were placed in the oven for one hour at 65oC, and deparaffination was followed by dipping them in xylene solution for ten minutes. They were then hydrated through a descending series of alcohol solutions (100%, 100%, 96%, 76%). Sections were stained with hematoxylin for five minutes and rinsed in tap water for five more minutes. For the partial discoloration of hematoxylin, a 1% differentiation solution was used for one second. Sections were stained with eosin for one minute, dehydrated in ethanol for five minutes, and cleared in xylene for another five minutes. Finally, the slides were covered with “Canada balsam” for light microscopical analysis [26]. In the present study, DAPI nuclear staining was used to investigate whether salicylate exposure could induce morphological alterations to cochlear hair cells.

The other seven positively charged slides were studied immunohistochemically, with anti-TGF-β1 [Santa Cruz, dilution 1:50] and anti-IL-6 [Santa Cruz, dilution 1:100] antibodies. The intensity of staining was evaluated by a semiquantitative scoring, based on a cross-count scale, according to the following categories: negative – no cross (-), mild – one cross (+), moderate – two crosses (++), intense – three crosses (+++). All slides were examined with brightfield microscopy by at least two independent observers, blinded to the identity of the immunohistochemical preparations.

Cochlear slices were prepared according to Jagger et al. (2000). The half-head was dissected in chilled slicing solution (4 °C) to remove the temporal bones. Because the slicing procedure damages the cochlea, the morphology of the sensory epithelium was protected by the introduction of a gel within the scalae. This was achieved by infusing a solution of artificial perilymph containing 35% pluronic gel at 4 °C via a glass micropipette inserted in the round window niche, with the infused solution flowing out of the cochlea through the opened oval window. The cochlea was then carefully oriented to obtain planar slices in the modiolar plane that radially transected the three scalae. The whole cochlea slicing was done in a slicing solution at room temperature (which solidifies the pluronic gel), and several slices of 250 − 300 mm thickness were obtained. Cochlear slices were then transferred to the chilled oxygenated slicing solution to dissociate the pluronic gel from the cochlear tissue and laid to recover for 15 min. The slice preparations were then mounted in a recording chamber perfused at 1 ml/min with warmed (23 °C) and oxygenated (95% O2, 5% CO2) recording solution.

Hair cells were labelled with Alexa-488 conjugated phalloidin and auditory nerve fibers were immunolabeled with monoclonal antibody targeting class III β-tubulin, as suggested in previous studies [27, 28].

### Statistical Analysis

The statistical program R (Version 4.1.2) was used for statistical analysis. DPOAE amplitudes for each experimental group were compared at each different time point separately (1st, 2nd, or 3rd week).

First, a gross analysis was attempted. The average DPOAE amplitudes were calculated for each frequency using data from all the rats in a specific experimental group. Then the average DPOAEs from all frequencies were calculated for each group and used in a one-way ANOVA or Kruskal-Wallis test, as well as in the following post hoc tests or pairwise comparisons.

Moreover, a more detailed and thorough analysis was carried out as well, in which the average DPOAEs for each group were compared at each different frequency (frequency-level analysis).

## Results

The animals each required four to seven sessions of 10 trials to be conditioned. All animals responded correctly to the conditioning stimulus (sound) during the last three training sessions with scores of at least 80%. The entire conditioning procedure required 2 or 3 d. Score, and false positive responses were then measured on 9 consecutive days, and the animals received daily intraperitoneal injections, as specified for each group in the study protocol.

### DPOAE amplitudes – gross Analysis

Differences in cochlear activity between groups were investigated using the corresponding DPOAE amplitudes. Our measurements showed a decrease in them. In the 1st week of our experiment, a significant difference in all frequencies examined was noted between group C and groups A, B, D, and E respectively (p < 0.001), as anticipated since group C was given double the dose of intraperitoneal salicylate (600 mg/kg/d).

At the next measurement, namely in the 2nd week, a significant difference was noted between group A and groups B, C and D, indicating that the damage caused by salicylate was clearly affecting cochlear activity in all groups in which it had been administered (p < 0.001). However, no differences between group D and groups B or C were found, suggesting a weak effect of memantine. Similarly, experimental groups B, C and D all differed significantly from group E (p < 0.001). DPOAEs in the Noiser group were not significant from those recorded in the Control group (p = 0.9).

**Fig. 1:**
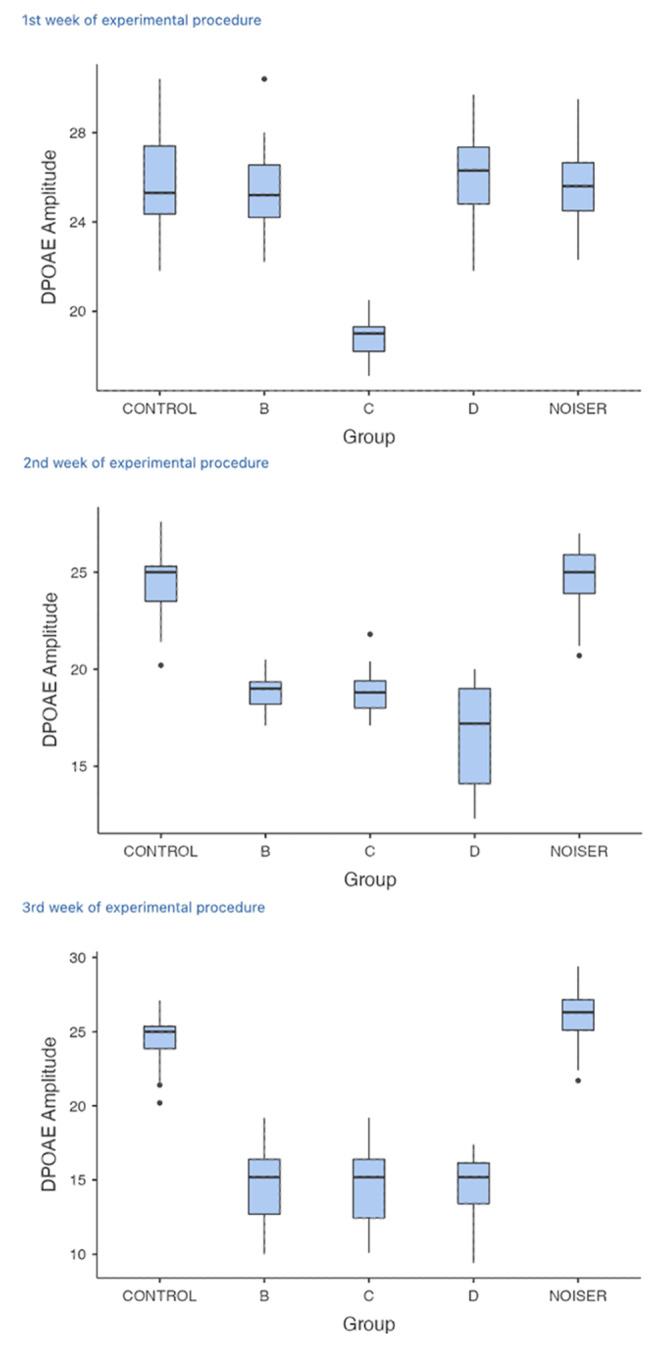
Average DPOAE amplitude for each group at the 1st, 2nd and 3rd week of the experimental period

At the 3rd measurement (3rd week), the same differences as the 2nd measurement were found. Groups B, C and D differed significantly from the Control and Noiser group (p < 0.001), but no more differences between these three experimental groups were noted, suggesting no effect of memantine. DPOAEs in the Noiser and Control groups did not differ significantly (p = 0.37). Figure 1 presents a box plot of the DPOAEs in each group at different measurements.

**Fig. 2:**
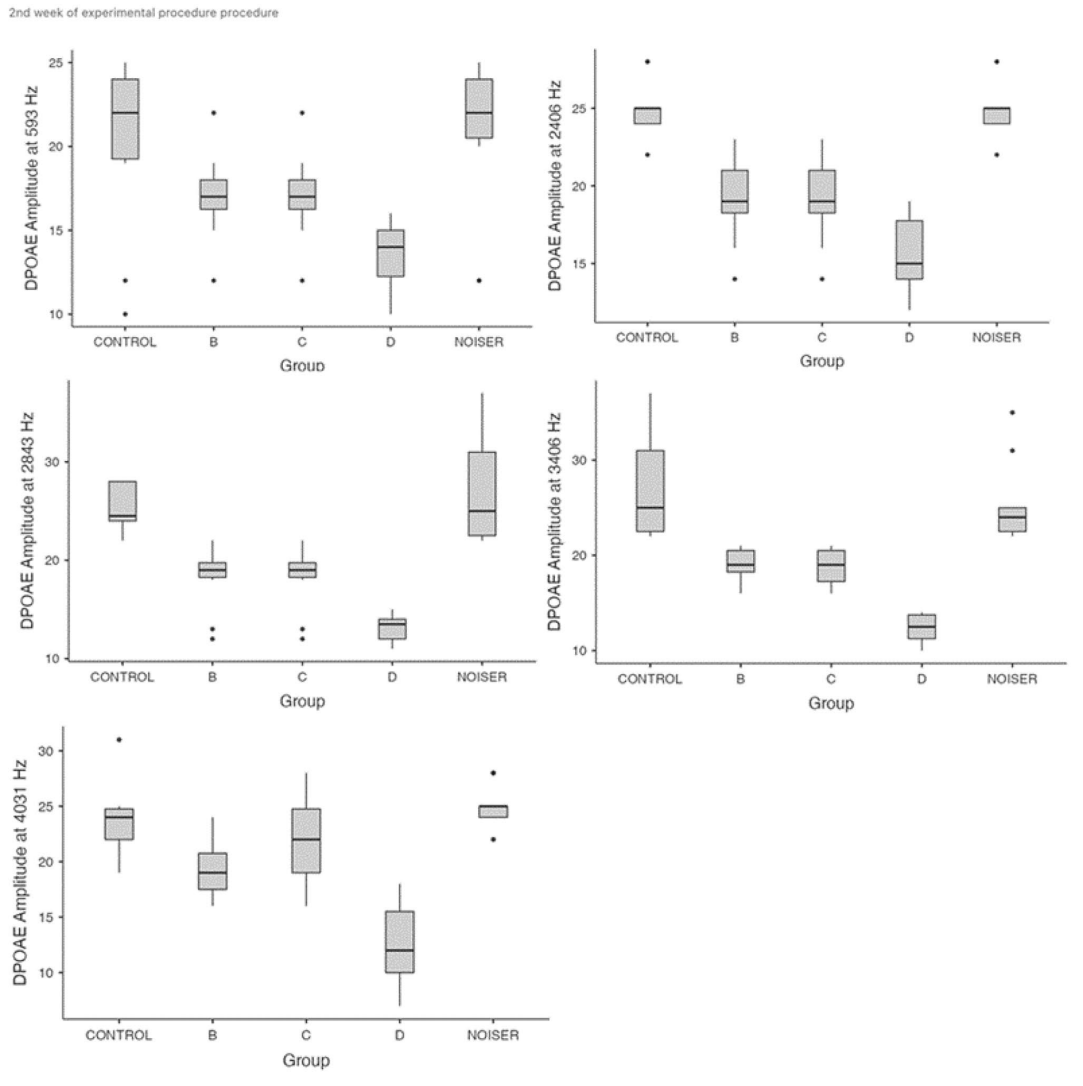
Frequency-level analysis of the DPOAE amplitude from the 2nd week of the experimental period. Significant difference was detected between group D and groups B, C at 593 Hz (p = 0.02), 2406 Hz (p = 0.01), 2843 Hz (p = 0.005), 3406 Hz (p = 0.001) and 4031 Hz (p < 0.001). The results were not in favor of group D, in which memantine was administered

Wanting to refine our statistical analysis for individual frequencies further, we will see that statistically significant differences are observed during the second week of the experiment. Specifically, a significant difference was detected between group D and groups B, C at the frequencies 593 (p = 0.02), 2406 (p = 0.01), 2843 (p = 0.005), 3406 (p = 0.001) and 4031 (p < 0.001). The results were however not in favor of group D, in which memantine was also administered. It should also be noted that DPOAE analysis of frequencies 718 and 843 in the 2nd week, contrary to the gross analysis, did not show significant differences between the groups (Fig. 2).

**Fig. 3:**
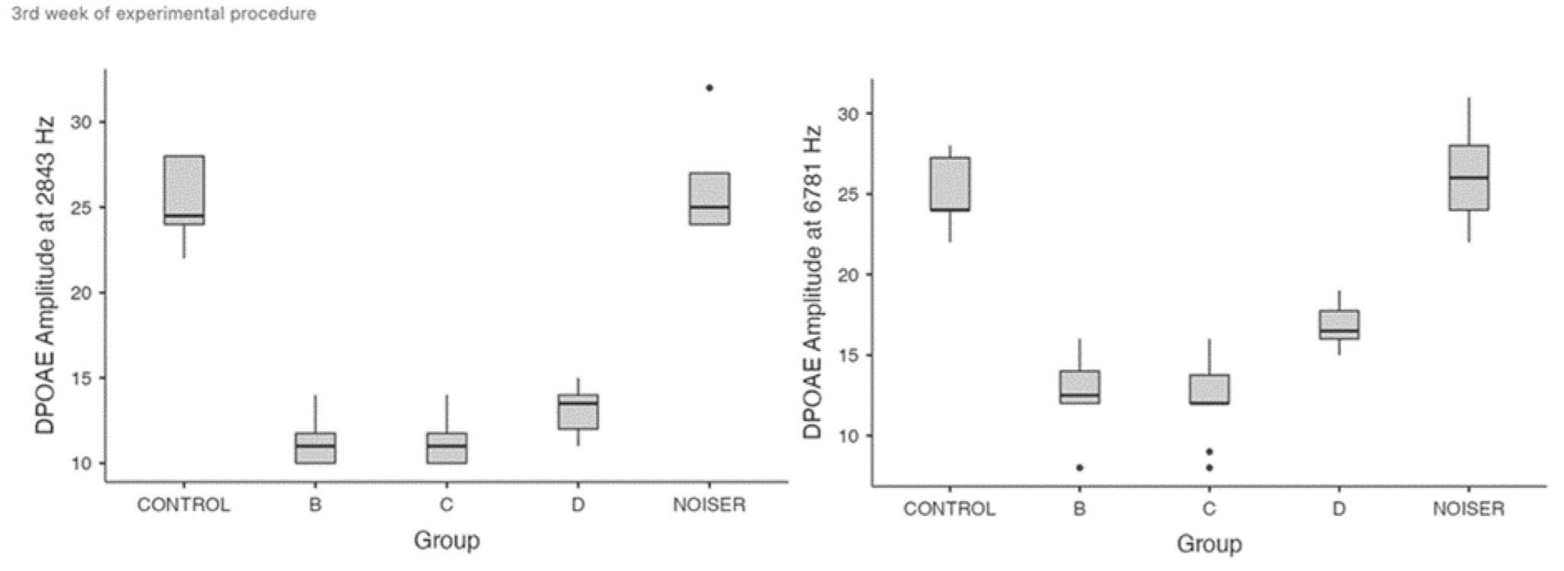
Frequency-level analysis of the DPOAE amplitude from the 3rd week of the experimental period. Statistical significance was found when comparing group D with groups B, C at 2843 Hz (p = 0.038) and 6781 Hz (p = 0.004)

At the 3rd measurement, statistical significance was found when comparing group D with groups B, C at the frequencies 2843 (p = 0.038) and 6781 (p = 0.004). Interestingly though, this time the results favored group D, highlighting a possible protective effect of memantine (Fig. 3).

### Histological Examination

Οbservations focus on Reisner’s membrane, outer and inner hair cells, and the spiral ganglion.

Light microscopic examination of eosin-hematoxylin-stained sections in Group A, did not reveal any major histopathological changes in Reisner’s membrane, while the immunohistochemical stainings for TGF-β1, as well as IL-6 markers, were found negative in all samples examined. In Group B, light microscopic examination of eosin-hematoxylin-stained sections revealed light detachment of the cells’ vestibular (Reisner’s) membrane with flattening in only 2 animals. (Image 4) All samples showed the immunohistochemical staining for the IL-6 and TGF-β1 markers as intense (+++). In Group C, eosin-hematoxylin staining did not reveal any pathological changes in the integrity and architecture of the vestibular (Reisner’s) membrane. Regarding the immunohistochemical staining for IL-6, it was interpreted as negative in all samples. The study of the TGF-β1 staining is in progress. Still, the first results indicate that TGF-β1-mediated apoptosis is not a possible mechanism of the observed lesions. In Group D, eosin-hematoxylin staining revealed light detachment of the vestibular (Reisner’s) membrane with mild shrinkage of the cellular cytoplasm (Image 5). The histological findings of the last group (Group E), which has been exposed only to noise, have shown no histological deformity, compared to the findings of the rats of the control group (Group A).

**Image 1:**
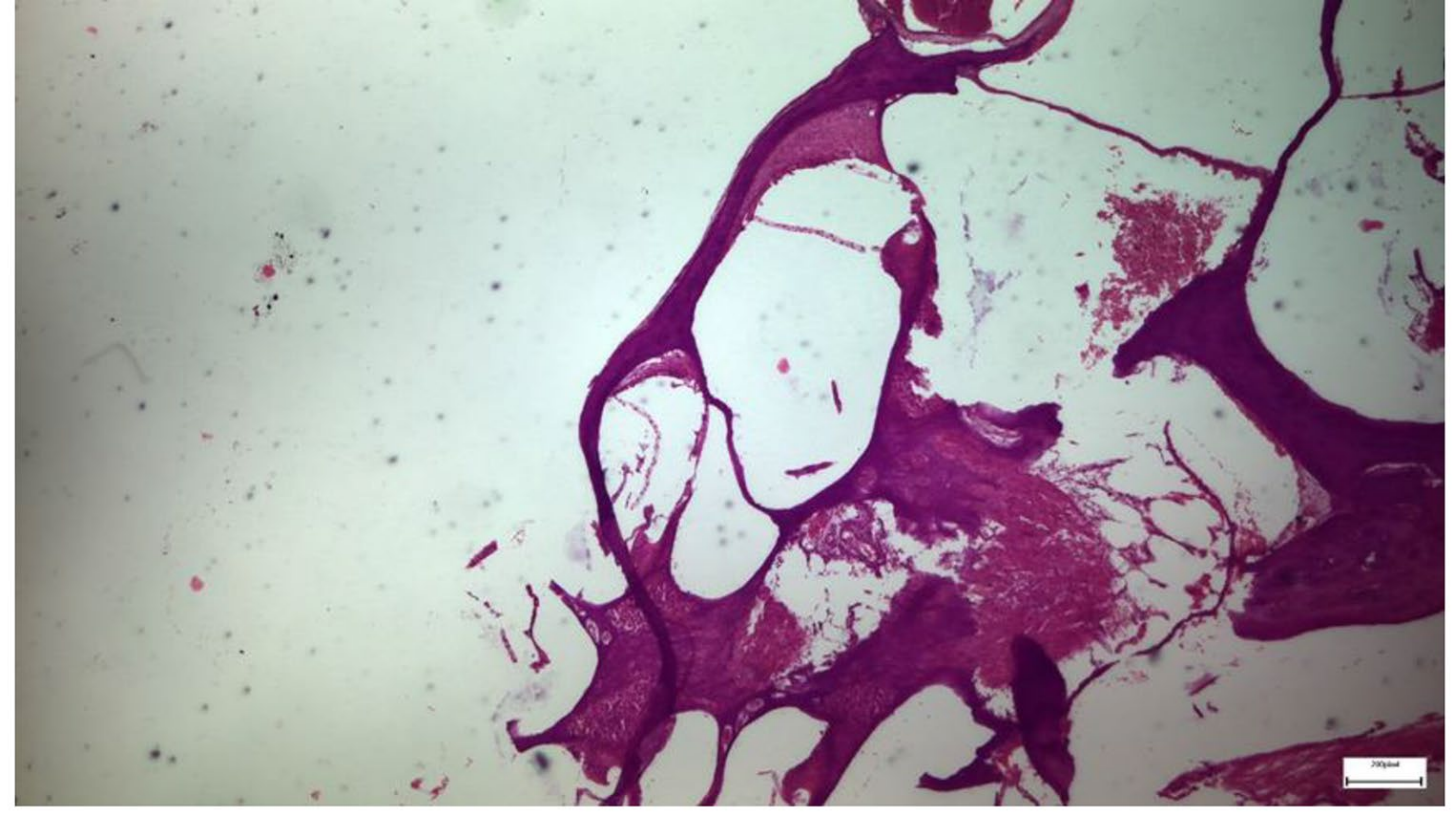
Group B specimen. Detachment of Reissner’s membrane with flattening of the cells. Intense eosin-hematoxylin staining is also to be mentioned

**Image 2:**
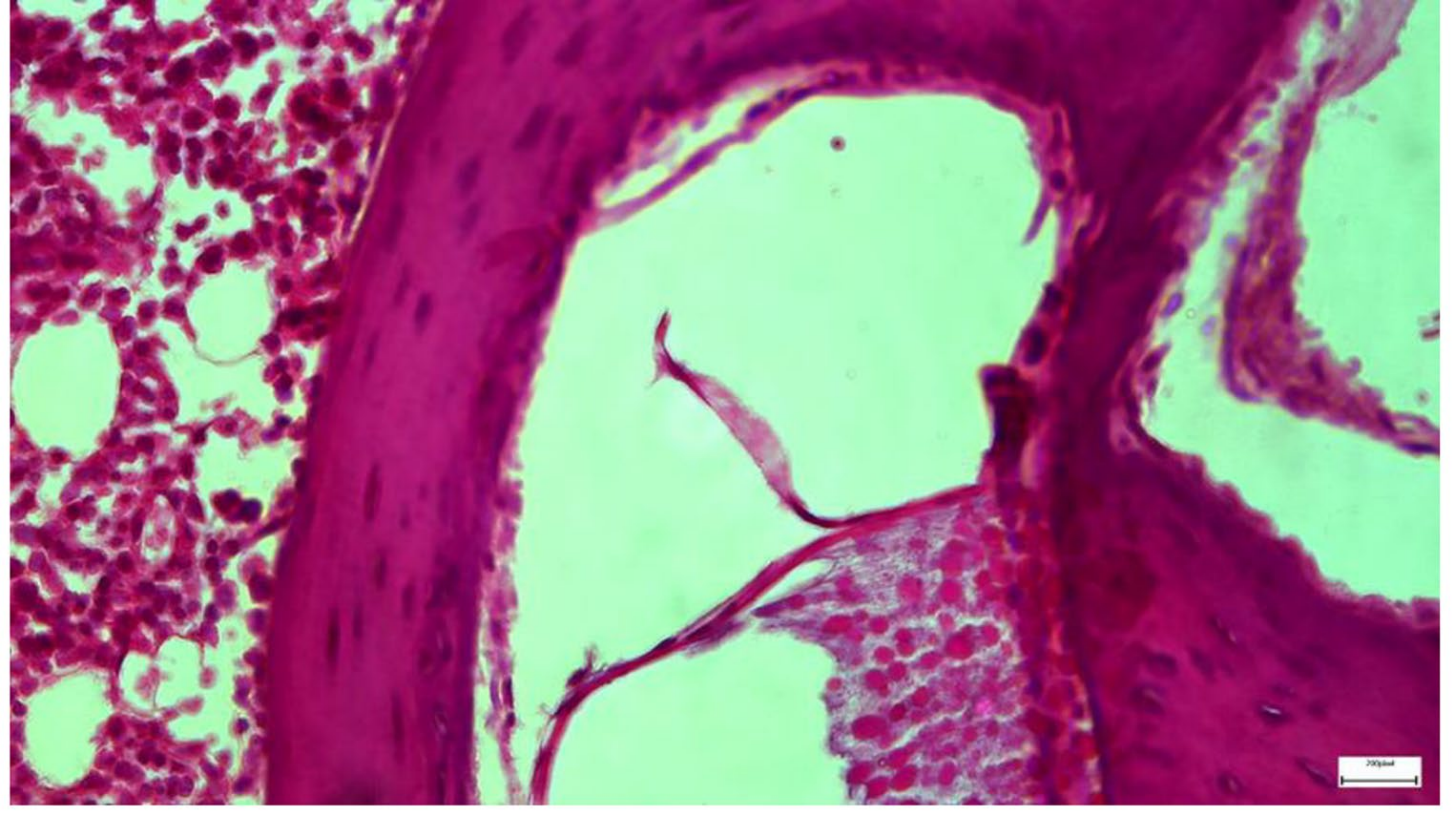
Group C specimen. Preservation of the integrity and architecture of the vestibular (Reissner’s) membrane with minor flattening of the cells after eosin-hematoxylin staining. Eosin-hematoxylin staining did not reveal any pathological changes in the integrity and architecture of the vestibular (Reissner’s) membrane but with minor flattening of the cells

OHCs and IHCs exhibited normal morphology as indicated by the nuclei of the hair cells throughout the period of salicylate exposure. This result suggested that cochlear hair cells are not affected by salicylate exposure. On the contrary, a decrease of the peripheral fibers projecting out from the spiral ganglion in all animals, which have been treated with salicylate, was observed. Memantine seemed to have played only a minor protective role.

### Behavioral Tests

In the present study, the animals treated only with salicylate (receiving either 300 mg ⁄ kg or 600 mg ⁄ kg per day) showed a significant increase in the number of false positive responses in an active avoidance task that is a surrogate measure for tinnitus. The number of the false positive responses was almost the same (p > 0.05), indicating that higher concentration does not seem to be related with more intense or troubling tinnitus. On the contrary the rats which received both salicylate and memantine showed a smaller number of false positive responses compared to the previously reported groups (p = 0.04), compared to groups B and D. Interestingly, the reactions of group E were the same as those of groups B and C.

## Discussion

The main findings of this study are the following:

- The greater the concentration of administered salicylate is, the greater the changes in auditory perception detected, either in intensity or in more frequencies, as recorded using DPOAEs.
- Administration of memantine does not have the potential to limit salicylate ototoxicity in the functional level (DPOAEs). This becomes evident both from the study of otoacoustic emission findings and from behavioral tests.
- Memantine appears to have a limited protective role in cochlear structures.
- Rats exposed to continuous sound (Group E) developed tinnitus, as evidenced by behavioral tests. In contrast to the experimental animals of groups B, C, and D, where changes in findings of otoacoustic emissions were observed, in this group no changes in auditory perception were observed.

It has been suggested that tinnitus can arise from damage at any level of the auditory pathway, but in most cases, it is cochlear structures that are damaged (26–28). These lesions can result in abnormal neuronal activity in the central auditory pathways. Among the suggested possible mechanisms are increased firing rate and neural synchrony [26–28].

Aspirin, a widely used non-steroidal anti-inflammatory medicine, can induce ototoxicity, including reversible tinnitus and hearing loss. Salicylate, the major active component of aspirin, also increases NMDA receptor responses in cochlear Sect. [27]. These findings imply that NMDA receptors, not the non-NMDA receptors, may be the major target of salicylate-induced ototoxicity in spiral ganglion neurons (SGNs) [28–30].

The site of generation of tinnitus is a key issue in understanding the mechanisms of the symptoms and the effectiveness of potential treatments. Together with others [31] we reported a slight salicylate-induced abolishment of DPOAEs. This confirms that salicylate-induced hearing loss is attributable to an effect on OHCs [32–34], without any dramatic change in their morphology or their structure, as observed in histological examinations. The above findings contradict those of relatively recent research of Cui et al. [35]. The results of these authors show that administration of salicylate improved DPOAE amplitudes during treatment. As it has been already stated above, in this study the rats received amikacin for 7 days. Yi et al. suggested that by administering salicylate for a longer period (14 days), they have noticed increased amplitudes of DPOAEs, which are mainly caused by increased outer hair cell (OHC) electromotility, enhancement of the average spectrum of electrophysiological cochleoneural activity, and induced changes to the synaptic ultrastructure of the dorsal cochlear nucleus (36). The same authors suggest that DPOAEs decreased after a single salicylate injection. The findings of the present study contradict the above mentioned data, as it has been shown that even 1 week after the beginning of the treatment the amplitude of DPOAEs seems to be reduced in specific frequencies.

Salicylate has been reported to increase the spontaneous activity of the auditory nerve [10, 11] and to change the average spectrum of cochleoneural activity [34, 35]. The increased spontaneous activity has also been observed in the inferior colliculus [29, 32], and the auditory cortex. Cells from nonauditory structures physically adjacent to the inferior colliculus showed no change in spontaneous activity [36, 37]. The selectivity of salicylate influencing auditory pathways argues against the nonspecific action of salicylate on the nervous system. This supports the view that abnormal activities are specifically propagated within the auditory system, and these abnormalities may be erroneously interpreted as sound by higher auditory centers [3, 6, 9].

Although NMDA receptors are present in SGNs, their functions in auditory transmission remain unclear. It is considered that NMDA receptors do not participate in fast postsynaptic transmission, since cochlear perfusion of APV does not influence the cochlear potential [38, 39]. NMDA receptors regulate the number of AMPA receptors on the surface of SGNs to modulate synaptic efficiency [40].

It has been proposed that inflammatory responses occur in the inner ear under various damaging conditions, including overstimulation with noise [41–45], and cisplatin-induced ototoxicity [46]. However, an association between proinflammatory cytokines and tinnitus has rarely been reported. In chronic tinnitus sufferers, a relaxation training program can result in significantly decreased stress, anxious depression, anger, and tinnitus disturbance, paralleled by a reduction of TNF-a, but not IL-6 or IL-10 [42, 43].

Hwang et al. demonstrated that salicylate-induced tinnitus correlates with increased gene expression of TNF-a and IL-1b [47]. The tinnitus scores of salicylate-treated mice showed significant positive associations with the expression levels of the TNF-a and IL-1b, and NR2B genes. Furthermore, the gene expression levels of TNF-a and IL-1b correlated positively with that of the NR2B gene.

An important point worth discussing is the implementation of the experimental model. A necessary element for conducting these experiments is the recognition on the part of the rats that they have tinnitus. Briefly, the behavioral indicator of tinnitus is determined by counting the number of false positive responses observed after the animals had been conditioned to respond to hearing a sound by displaying a motor task [45–47]. Animals were trained to jump on a climbing pole upon hearing a sound — the false positive responses being the number of climbs observed during silent periods. In contrast to other protocols in which animals are deprived of food or water — in some cases leading to major loss of body weight [32–48] - this paradigm did not involve changes in the animals’ physiological state. This presented great advantages, especially when surgical procedures are required to administer drugs locally (i.e., directly in contact with cochlear fluids through the round window membrane) to the question of the site of generation of tinnitus induced by salicylate. Since inner ear hair cells use glutamate as a neurotransmitter to convey auditory information to the brain, glutamate antagonists block salicylate-induced tinnitus.

Another interesting finding, based on the behavioral tests and their results, is that the rats exposed to constant noise presented with tinnitus without any simultaneous hearing loss. In humans, the prevalence of tinnitus has been reported to increase with increasing hearing loss in noise-exposed workers that were routinely screened (48–50], whereas, in those claiming compensation for work-related hearing loss, the prevalence of tinnitus was approximately constant over a wide range of hearing [48]. Though experimental, the results presented in this article suggest that tinnitus can occur without previous hearing loss.

In the present study, the possibility of cochlear dysfunction as a cause of tinnitus was studied using DPOAEs, based on its current use as an efficient tool for the objective evaluation of the inner ear function, especially of the outer hair cells of the cochlea [45–47]. One should also note some similarities and differences in the findings and the conclusions between those of this study, where rats have been examined, and those of previous studies which have examined the course of hearing loss and occurrence of tinnitus in humans. Previous authors have suggested that hearing loss is a risk factor for tinnitus, and even tinnitus patients with normal audiograms might have restricted cochlear damage (57–60). It has also been suggested that not all patients with hearing loss develop tinnitus [32, 46–48]. Given that the present study examined animals, one should mention that all animals exposed to noise that developed tinnitus did not suffer from hearing loss in any frequency. Moreover, the histological examination of their cochlea showed no destruction of their inner ear structure.

At that point, the possibility of structural or other changes in the function of the brain and its auditory cortex should be addressed. Previous experimental studies suggested substantial cortical reorganization [32, 47, 48]. It has been suggested that tinnitus can arise from damage at any level of the auditory pathway, but most cases are caused by cochlear damage. These lesions can result in neuroplasticity alterations in the peripheral and central auditory systems [1, 2, 49, 32–48].

## Conclusions

This study shows that the administration of memantine does not contribute substantially to the reduction of tinnitus. An important element to point out is that the emergence of tinnitus, in rats exposed to continuous sound (16 kHz from a sound generator), is not a result of loss of auditory perception, at least not in the first stages after the exposure to intense noise.

## Data Availability

the data presented in this study are available on request from the corresponding author.

## Declarations

### Statements and Declarations

Ethics Approval the study was conducted according to the quidelines of the Declaration of Helsinki and approved by the local ethics committee (Medical School) for animal research (decision nr 537,227/2465) in Aristotle University Thessaloniki, Greece.
